# *Mycobacterium goodii* endocarditis following mitral valve ring annuloplasty

**DOI:** 10.1186/s12941-017-0190-4

**Published:** 2017-03-21

**Authors:** Rohan B. Parikh, Matthew Grant

**Affiliations:** 1 Texas Heart Institute, 6770 Bertner Avenue, Houston, Tx 77030 USA; 2 Yale School of Medicine, 333 Cedar St, New Haven, CT 06510 USA

**Keywords:** *Mycobacterium goodii*, Endocarditis, Gene sequencing, Prostheses related infections

## Abstract

**Background:**

*Mycobacterium goodii* is an infrequent human pathogen which has been implicated in prosthesis related infections and penetrating injuries. It is often initially misidentified as a gram-positive rod by clinical microbiologic laboratories and should be considered in the differential diagnosis.

**Case presentation:**

We describe here the second reported case of *M. goodii* endocarditis. Species level identification was performed by 16S rDNA (ribosomal deoxyribonucleic acid) gene sequencing. The patient was successfully treated with mitral valve replacement and a prolonged combination of ciprofloxacin and trimethoprim/sulfamethoxazole.

**Conclusion:**

Confirmation of the diagnosis utilizing molecular techniques and drug susceptibility testing allowed for successful treatment of this prosthetic infection.

## Background

*Mycobacterium goodii* is a rapidly growing non-tuberculous mycobacterium (NTM) belonging to the *Mycobacterium smegmatis* [1] group. Its importance has become increasingly appreciated as a pathogen over the last 20 years, with a predilection towards infecting tissues at the site of penetrating injuries. Antibacterial treatment strategies against this pathogen are diverse but reported case cure rates are high. Here we describe what the authors believe to be the second reported case of *M. goodii* endocarditis ever reported (first time involving a ring annuloplasty).

## Case presentation

A 67-year-old Caucasian man, retired financier, with a history of severe mitral regurgitation status post ring annuloplasty repair complicated by right sided hemothorax requiring two reoperations to achieve hemostasis, presented to an outside hospital 3 weeks postoperatively with fever, loss of appetite, and gait disturbance.

**Fig. 1:**
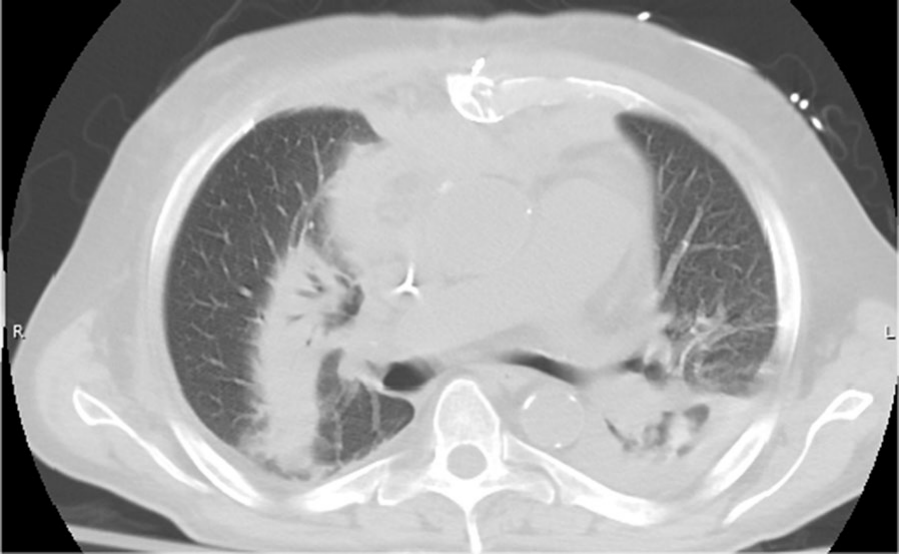
Computed tomography of chest showing bilateral infiltrates and bilateral pleural effusion

On examination the patient vital signs were normal, lungs were clear, a mild 1/6 systolic murmur was appreciated at the apex, and a drain was in place for a groin seroma related to recent left heart catheterization. He had an unsteady gait and exhibited mild left lower extremity weakness (4/5). His brain magnetic resonance imaging showed multiple ring-enhancing lesions in the pons and posterior fossa suggestive of septic emboli. Transthoracic echocardiography showed moderate mitral regurgitation without any vegetation. Blood cultures grew gram-positive rods suspicious for *Actinomyces* spp. and he was started on vancomycin and ampicillin/sulbactam. He developed a morbilliform cutaneous eruption felt to be related to the ampicillin and was switched to vancomycin/ceftriaxone. A computed tomography scan of the chest (Fig. 1) was done which showed bilateral infiltrates and mild pleural effusions.

**Fig. 2:**
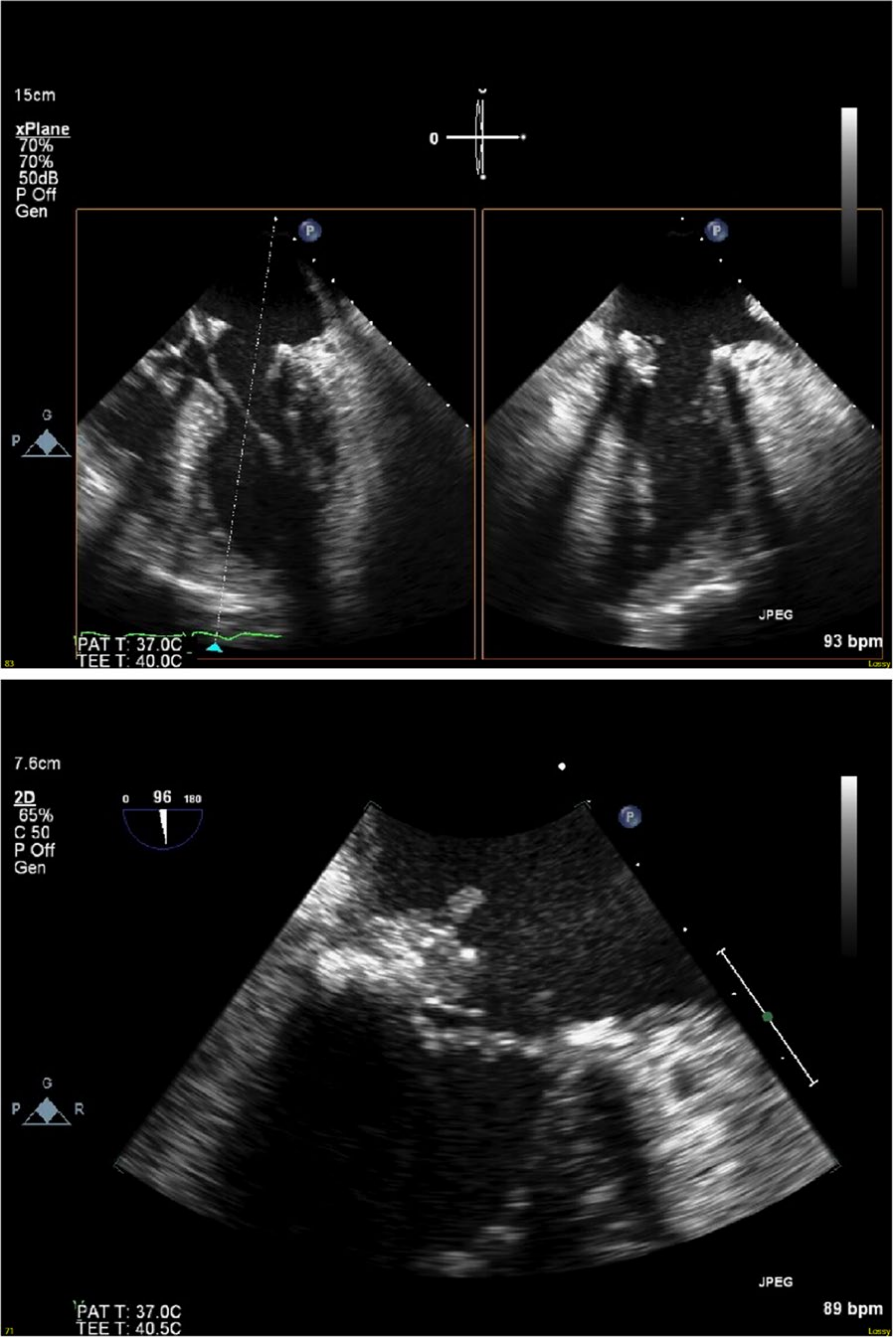
Trans-esophageal echocardiogram showing mitral valve vegetation in the mid-esophageal plane

The patient was then transferred to our hospital on day 14 for further management. A transesophageal echocardiogram (TEE) (Fig. 2) showed vegetations on the P3 annulus and evidence of ring dehiscence in A2, A3, and P3 areas. He completed penicillin desensitization and was successfully narrowed to penicillin G to be optimally treated for presumptive actinomycotic endocarditis and both vancomycin and ceftriaxone were stopped. On hospital day 18 he underwent mitral valve replacement with a bioprosthetic valve (27 mm. St. Jude prosthesis). Intraoperatively, vegetations were confirmed on the mitral valve and tissue cultures from the explanted native mitral valve suggested a rapid growing mycobacterium rather than an Actinomyces spp. Empiric treatment with meropenem (1 g IV thrice a day)/amikacin (1 g IV per day)/clarithromycin (500 mg oral twice a day)/ciprofloxacin (400 mg IV twice a day) was initiated pending final confirmation and susceptibility testing. On day 21 it was confirmed that the gram-positive isolate was a 100% match to the *M. goodii* strain (American Type Culture Collection or ATCC #700504) using 16S sequencing (MicroSeq 500 bp 16S rDNA kit), but *M. smegmatis* could not be ruled out due to a high-level sequence homology [99.5% match to *M. smegmatis* type strain (ATCC 19420) with two mismatches]. Serial blood cultures sterilized on hospital day 26 and he was transferred to a nursing facility after repeat TEE showed a normally functioning prosthetic mitral valve.

On the day following discharge into the rehabilitation facility, the patient developed a maculopapular eruption involving his flanks and back, which progressed to involve his anterior trunk and all four limbs. He presented to the infectious diseases clinic 3 days after discharge where the rash was suspected of being related to the meropenem and tigecycline was substituted; however, he had an acute anaphylactoid reaction (involving dyspnea and hypotension) during the loading dose of tigecycline (100 mg once) which was subsequently replaced with linezolid (600 mg oral twice a day). His susceptibility reports returned which showed the mycobacterium was susceptible to trimethoprim–sulfamethoxazole, amikacin, doxycycline, ciprofloxacin, imipenem, linezolid and resistant to clarithromycin. In accordance, his regimen was changed to ciprofloxacin (500 mg oral twice a day)/trimethoprim–sulfamethoxazole (1 DS tablet oral twice a day).

The patient was ultimately treated with a total of 6 months of therapy. He followed up in infectious diseases clinic on days 61 and 135 and was contacted by phone 137 weeks post valve replacement and there were no complaints or signs of intervening relapse, was highly active and back to all his prior recreational activities.

## Discussion

A broad literature search was done from PubMed, Scopus and OvidSP databases containing the search terms *M. goodii* to try and identify all human infections with this organism. *M. goodii* was proposed as a new rapidly growing species related to *M. smegmatis* based on gene sequencing work by Brown et al. [1] in 1999 in continuation to the work done by Wallace et al. [2]. We have summarized to the best of our knowledge all the published reports about *M. goodii* infections (Table 1) after Brown [1]. A total of 45 cases (including our patient) have been reported to date. Eleven (25%) cases were wound/bone infections due to trauma. Twenty-two (49%) cases were iatrogenic, with eighteen (38%) involving infection of prosthetic materials. Eight (18%) cases were pulmonary, which were strongly associated with histological findings of lipoid or granulomatous pneumonias. Four (9%) cases had unclear clinical diagnosis but were confirmed to be *M. goodii* microbiologically.

**Table 1:** Selected *M. goodii* reported human cases after Brown et al.

Infection type	Age	Sex	Comorbidities	Treatment	Microbial susceptibility
Mitral valve endocarditis complicating ring annuloplasty	67	M	None	Mitral valve replacement, ciprofloxacin + TMP/SMX (6 months)	Susceptible to TMP/SMX, amikacin, doxycycline, ciprofloxacin, imipenem, linezolid Resistant to clarithromycin
Prosthetic mitral valve endocarditis [8]	76	F	None mentioned	Mitral valve replacement, tigecycline + ciprofloxacin (2 weeks) doxycycline + ciprofloxacin (11 weeks)	Resistant to cefotaxime, erythromycin, clarithromycin
Pacemaker site infection [9]	23	M	Partial AV canal defect repair at 5 years of age	Pacemaker retained, ofloxacin + amikacin (1 month), doxycycline (6 months)	Resistant to clarithromycin
Pacemaker pocket infection [10]	85	M	Ischemic cardiomyopathy	Pacemaker replacement, TMP/SMX (8 weeks)	Susceptible to TMP/SMX, minocycline, imipenem
Pacemaker pocket and bloodstream infection [11]	82	M	None mentioned	Pacemaker removal, amikacin + minocycline	Resistant to clarithromycin, cefoxitin, clindamycin, vancomycin
Pacemaker pocket infection [12]	74	F	Bicuspid aortic valve, hypothyroidism, diabetes mellitus, gastro-esophageal reflux disease, asthma	Pacemaker removal, ciprofloxacin + doxycycline (6 months)	Susceptible to amikacin, ciprofloxacin, doxycycline, imipenem, linezolid, tobramycin and trimethoprim/sulfamethoxazole Resistant to clarithromycin
Ventriculo-peritoneal shunt infection [13]	60	F	Allergy to sulfa drugs	Ventriculoperitoneal shunt removal, imipenem + moxifloxacin (5 weeks), moxifloxacin monotherapy (3 additional months)	Resistant to azithromycin, clarithromycin
Hernia mesh infection with abdominal wall abscess [14]	65	M	None mentioned	Mesh removal, TMP/SMX	Resistant to cefoxitin, clarithromycin
Hernia mesh infection [15]	64	M	None mentioned	Patch removal. Antibacterial treatment not specified	Susceptible to ciprofloxacin, doxycycline and TMP/SMX
Skin graft infection [16]	6	M	None mentioned	TMP/SMX + linezolid (6 months)	Resistant to clarithromycin, amoxicillin/clavulanate
Prosthetic total knee joint infection [17]	44	M	Nicotine dependence, significant ethanol use	Removal of prosthesis, TMP/SMX (stopped after 1 week by patient), minocycline + ciprofloxacin (6 months)	Resistant to clindamycin
Prosthetic total knee joint infection [15]	75	F	None mentioned	Prosthesis replaced	Susceptible to ciprofloxacin, doxycycline and TMP/SMX
Prosthetic total hip joint infection [15]	64	M	None mentioned	Prosthesis replaced	Susceptible to ciprofloxacin, doxycycline and TMP/SMX
Olecranon bursitis following corticosteroid injections [18]	60	M	Diabetes mellitus	Doxycycline + ciprofloxacin (10 weeks)	Resistant to clarithromycin
Postcataract endophthalmitis [19]	67	M	None mentioned	Pars plana vitrectomy + lens removal, intravitreal amikacin × 2 doses	None reported
Aspiration pneumonia [20]	15	F	Rickettsial infection	Laparoscopic Heller myotomy with fundoplication, ciprofloxacin + doxycycline (12 months)	Resistant to clarithromycin
Complicated parapneumonic effusion [21]	66	M	None mentioned	None mentioned	None mentioned

Since there is no available clinical trial or prospective data to guide therapy for this infection, we extrapolated a treatment approach from the accumulated experience with other more common rapid growing NTM species to treat our patient with this incredibly rare disease. Our empiric regimen selection was further made challenging by the development of a second drug eruption and an anaphylactoid infusional reaction, both of which required cessation of drugs and subsequent drug substitution.

## Conclusions

Rapid growing mycobacteria should be suspected in trauma or prosthetic related infections not responding to initial empiric therapies. Molecular techniques are rapid and reliable for confirmation of rapid growing mycobacterial infections and are recommended by the Infectious Diseases Society of America guidelines [3]. Once rapid growing mycobacteria are suspected, 16S ribosomal sequencing should be used if available for species level identification. 16S rRNA gene sequences contain hypervariable regions that can provide species-specific signature sequences useful for identification of bacteria [4]. Since *M. goodii* has the ability to form biofilms [5], prosthesis removal is indicated to achieve cure if feasible. Macrolides should not be included in the empirical/definitive treatment since it has been shown that the organism has intrinsic macrolide resistance conferred by novel rRNA methylase genes *erm*(38) and *erm*(39) [6, 7]. This has also been seen widely in the susceptibility testing for the organism. The organism is usually susceptible to sulfonamides, amikacin, doxycycline, imipenem, fluoroquinolones and they should be optimized for dose and duration according to the severity and comorbidities.

## Abbreviations

ATCC: American Type Culture Collection
rDNA: ribosomal deoxyribonucleic acid
TEE: transesophageal echocardiogram
